# *Helicobacter pullorum*: An Emerging Zoonotic Pathogen

**DOI:** 10.3389/fmicb.2017.00604

**Published:** 2017-04-10

**Authors:** Sundus Javed, Farzana Gul, Kashaf Javed, Habib Bokhari

**Affiliations:** Department of BioSciences, COMSATS Institute of Information TechnologyIslamabad, Pakistan

**Keywords:** *Helicobacter*, enteric *Helicobacter* species, *H. pullorum*, zoonotic pathogen, foodborne pathogen

## Abstract

*Helicobacter pullorum* (*H*.pullorum) commonly colonizes the gastrointestinal tract of poultry causing gastroenteritis. The bacterium may be transmitted to humans through contaminated meat where it has been associated with colitis and hepatitis. Despite the high prevalence of *H. pullorum* observed in poultry, little is known about the mechanisms by which this bacterium establishes infection in host and its virulence determinants. In this article we aim to provide an overview of this emerging zoonotic pathogen; its general characteristics, hosts, prevalence, and transmission as well as its pathogenic potential. We also discuss possible control strategies and risk of disease emergence.

## Introduction

*Helicobacter pullorum* (*H. pullorum*) was first discovered by Stanley in 1994. He reported *Campylobacter*-like organisms in the liver, duodenum and caecum of chickens, as well as humans suffering from gastroenteritis. Due to its unique DNA homology and total protein electrophoretic patterns, it was classified as a novel species belonging to the *Helicobacter genus* (Stanley et al., 1994). The bacterium is an important member of the enterohepatic *Helicobacter* species (EHS) which predominantly colonize the intestine and the hepatobiliary system of the host (Hameed and Sender, 2011). In the following review we aim to provide a comprehensive overview of *H. pullorum* prevalence, its associated pathology as well as reported virulence and antibiotic resistance mechanisms.

## General characteristics

*H. pullorum* is a gram-negative bacterium, slightly curved rod in shape, with a single polar flagellum which is non-sheathed. It is a motile, non-spore forming, microaerophilic bacterium, which best grows at 37–42°C (Hassan et al., 2014a). *H. pullorum* produces catalase, reduces nitrates, but lacks urease, indoxyl acetate esterase, or alkaline phosphatase activity.

Genome Sequence information from 5 *H. pullorum* strains including one human strain (MIT 98-5489) isolated from a patient suffering from gastroenteritis and four poultry isolates (229334/12, 229336/12, 229254/12, 229313/12) are available at the NCBI database. The database also includes plasmid sequences from 2 strains. The genomic DNA has 33% GC content with a 1,919 kb circular chromosome coding for 2,044 genes of which 2008 are protein coding (Shen et al., 2014).

## Lipopolysaccharides (LPS)

Structural characterization of *Helicobacter pullorum* purified lipopolysaccharides (LPS) using electrophoretic, serological, and chemical methods reveals O-polysaccharide chain bearing lipopolysaccharides. 3-hydroxytetradecanoic acid and 3-hydroxyhexadecanoic acid are important components of *H. pullorum* LPS with low variability between chicken and human isolates. The bacterium exhibits high hydrophilicity, therefore water based extraction instead of acid glycine is considered to be more effective. *H. pullorum* LPS has the highest relative *Limulus* amoebocyte lysate activity of all *Helicobacter* species lipopolysaccharides, indicating high endotoxin activity (Hynes et al., 2004). Polysaccharides of *H. pullorum* may play an important role in bacterial adhesion since competitive binding of sulphated groups of heparin results in marked reduction in host cell adhesion (Lutay et al., 2011). Ability of *H. pullorum* LPS to induce nuclear factor-Kappa B activation in host cells may play an important role in inflammation leading to the gastroenteritis observed in *H. pullorum* infection (Hynes et al., 2004).

## N-linked glycosylation system

Bacterial N-linked glycosylation system was discovered in *Campylobacter jejuni*. Oligosaccharyltransferase PglB is the key enzyme of this system involved in the coupling of glycan to asparagine residues of the glycoprotein. Until now, all characterized *Helicobacter* species lacked *pgl* genes except *H. pullorum, H. canadensis*, and *H. winghamensis*.

*H. pullorum* possesses two unrelated pglB genes (pglB1 and pglB2), neither of which is located within a larger locus like *C. jejuni*. PglB1 protein of *H. pullorum* displays oligosaccharyltransferase activity in complementation experiments. On the other hand pglB2 lacks oligosaccharyltransferase activity *in vitro*. Moreover, insertional knockout mutagenesis of pglB2 gene proved lethal for the bacterium suggesting that it is essential for its survival (Jervis et al., 2010). N-linked glycosylation is common in eukaryotes but rarely seen in bacteria. The description of N-linked glycosylation in another bacterial system presents an interesting opportunity for protein glycoengineering and possibilities for future therapeutic applications.

## Survival and transmission

Catalase enzyme plays a crucial role in protection of *H. pullorum* against oxidative stress of host and environment (Sirianni et al., 2013). The bacterium is able to tolerate high bile stress and variation in expression of certain bile stress response proteins has been suggested (Hynes et al., 2003). In a report by Bauer and colleagues, the *H. pullorum* two-component system (TCS) was shown to be involved in the control of nitrogen metabolism by regulating the expression of glutamate dehydrogenase. *H. pullorum* TCS is composed of an AmtB ammonium transporter and a PII protein consisting of the HPMG439 and its cognate histidine kinase (HK) HPMG440 (Bauer et al., 2013). In this respect the bacterium resembles *C. jejuni* than *H. pylori*. Moreover, the ability of the bacterium to tolerate oxidative stress and live under high bile stress enables it to occupy various niches in the enteric system of the host including the gall bladder, as mentioned in subsequent sections.

## Prevalence

*H. pullorum* naturally infects many poultry birds, some rodent species as well as humans. Gastroeneteritis in farm raised birds, including chicken, turkey, and guinea fowl has been associated with *H. pullorum* infection. The infection has been linked to vibrionic hepatitis lesions in chickens (Burnens et al., 1994) and diarrhea in humans (Ceelen et al., 2005a). Meanwhile, natural infection of *H. pullorum* strains in rats and rabbits has also been reported (Van den Bulck et al., 2006; Cacioppo et al., 2012). *H. pullorum* prevalence reports from various regions have been summarized in Table 1.

**Table 1:** **Summary of published *H. pullorum* prevalence data in poultry**.

**Region**	**Source**	**Sample size**	**Identification**	**Prevalence**	**References**
Egypt	Poultry (Chickens, Turkey, Ducks)	1,800 (cloacal swabs, cecal swabs and liver)	16S rRNA gene PCR assay	60% (Chickens)	Hassan et al., 2014b
				0% (Turkeys)	
				0% (Ducks)	
Marmara Turkey	Broiler chickens	96 (cecum and colon)	16S rRNA gene PCR assay	55.21%	Beren and Seyyal, 2013
				32.29% (cecum)	
				10.15% (colon)	
				15.63% (both)	
Ardabil	Broiler chickens	120 (chicken with gastroenteritis)	Biochemical tests (oxidase, catalase, nitrate reduction, urease)	7.5% (cecum)	Shahram et al., 2015
				5% (liver)	
Iran	Human fecal samples	100 (gastroenteritis)		2.5% (Thigh)	
				6%	
	Broiler chickens	100 (cecum)	16SrRNA gene PCR assay	41%	Jamshidi et al., 2014
Belgium	Broiler chickens	110 samples from 11 flocks (gastrointestinal tract and liver)	16S rRNA gene PCR assay	33.6% (cecum)	Ceelen et al., 2006a
				31.8% (colon)	
				10.9% (jejunum)	
				4.6% (liver)	
Belgium	Human fecal samples	531 (Gastroenteritis)	16S rRNA gene PCR assay	4.3% (gastroenteritis)	Ceelen et al., 2005b
		100 (healthy individuals)		4.0% (healthy)	
Selangor Malaysia	Broiler and village chickens	89 samples, 32 (village). 57 (Broiler chickens)	16S rRNA gene PCR assay	24.72%	Wai et al., 2012
Libson	Chicken meat	17 samples	16S rRNA gene PCR assay	23.52%	Borges et al., 2015
Italy	Farms (broiler chicken)	169 ceca (30 conventional farms)	Genotyping analysis(AFLP, PFGE)	100% (conventional farms)	Manfreda et al., 2011
		39 ceca (8 organic farms)		100% (organic farms)	
		40 ceca (7 free-range farms)		57.1% (free-range farms)	
	Farms (Turkeys)	55 (cecum)	16SrRNA gene PCR assay	76.4%	Zanoni et al., 2011
	Broiler chickens and laying hens	60 (cecum)	16SrRNA gene PCR assay	100%	Zanoni et al., 2007

### Poultry

*H. pullorum* has been isolated from various poultry tissues. 76.4% of Turkeys were found to be infected with the bacterium in Finland whereas no bacterial growth in turkey, cloacal, cecal, and liver samples was observed in a report from Egypt (Zanoni et al., 2011; Hassan et al., 2014b). Meanwhile, in chickens variable prevalence rates have been reported from various regions. A Polish study depicted 23.5% fresh chicken meat samples from different producers to be positive for *H. pullorum* (Borges et al., 2015). Whereas, 57.1% free-range farm birds and 100% broiler, layer, and organic farm chickens were infected with *H. pullorum* in Italy (Zanoni et al., 2007; Manfreda et al., 2011). Bacterial isolates obtained from the gastrointestinal tract and liver of 110 broiler chickens in Belgium were tested through PCR where 33.6% (cecum), 31.8% (colon), 10.9% (jejunum), and 4.6% (liver) isolates tested positive for the bacterium (Ceelen et al., 2006a).

39.33% prevalence rate was observed in Egypt using a *H. pullorum* species-specific 16S rRNA PCR on isolates from 900 cloacal, cecal, and liver isolates of broiler chickens, while there was no bacterial growth from duck samples (Hassan et al., 2014b). A study spanning 32 villages in Selangor and Malaysia testing broiler chickens for culture and PCR based identification of *H. pullorum*, reported 24.72% prevalence rate, where 12.36% chickens were co-infected with *Campylobacter spp* (Wai et al., 2012). On the other hand a higher *H. pullorum* prevalence rate of 55.21% was reported from Turkey where 12 broiler chicken flocks were tested (Beren and Seyyal, 2013).

Meanwhile, in the province of Ardabil, Iran 120 samples of chickens with gastroenteritis were tested using biochemical tests for the identification of *H. pullorum*. Results obtained through this study showed 7.5, 5, and 2.5% *H. pullorum* prevalence rates in cecum, liver and thigh meat samples, respectively (Shahram et al., 2015). However, another study from Iran evaluated 100 cecal samples from the gastrointestinal tract of broiler chickens using PCR and observed 61% prevalence of *H. pullorum* (Jamshidi et al., 2014). Cecum seems to be the preferred niche of the bacterium with fewer prevalence rates in the liver.

As discussed earlier *H. pullorum* is associated with vibrionic hepatitis in chickens, although the evidence seems singular. Later studies do not find any particular link between colonization of the bacterium and macroscopic liver lesions (Burnens et al., 1996; Ceelen et al., 2005b). This can be explained by the fact that *H. pullorum* prevalence reported in the study was low and may be reflective of colonization with hypervirulent strains.

*H. pullorum* has the ability to contaminate the carcasses of the poultry and is considered a food borne pathogen (Mohamed et al., 2010). Overall it may be expected that the prevalence of *H. pullorum* is generally underestimated as noted by Wainø et al. (2003). Generally gross underestimation of prevalence rates may be expected when relying on phenotypic tests commonly employed for identification. This is may be expected since screening for *H. pullorum* is not undertaken and *H. pullorum* strains fail to thrive on the mCCDA medium employed in the laboratory to select for campylobacters. This underestimation is supported by PCR identification of *H. pullorum* from broiler chicken isolates that were originally denoted unspeciated cultures or falsely identified as *Campylobacter lari* (Wedderkopp et al., 2000; Wainø et al., 2003).

Presence of the bacterium in the gut of chickens may have a wider impact on the birds' gastrointestinal physiology than having a pathological outcome. As a recent study suggests that *H. pullorum* impacts the gastrointestinal microbiota of commercial broiler chickens, influencing the *Lactobacillus, Streptococcus, Ruminococcaceae* abundance as well as prevalence of *Corynebacterium* species in the chicken gut (Kaakoush et al., 2014). However, the overall impact on the birds' physiology and health (e.g., net weight gain) as well as susceptibility to infection remains to be investigated.

### Humans

*H. pullorum* is a zoonotic bacterium that has also been associated with certain enteric infections in humans. *H. pullorum* has been associated with recurrent diarrheal illness in patients after treatment suggesting the possibility of chronic infection (Steinbrueckner et al., 1997). Case of a 35 year old male suffering from *H. pullorum* induced bacteraemia, presented with abdominal pain along with profuse diarrhea has also been reported (Tee et al., 2001). In Iran, human diarrheal samples were evaluated for presence of *H. pullorum* with a 6% prevalence rate (Shahram et al., 2015). On the other hand a Belgian study showed 4.3% *H. pullorum* prevalence in fecal samples from patients with gastroenteritis compared to clinically healthy individuals (Ceelen et al., 2005a). In another study 158 fecal samples were collected from under-five children with diarrhea and 35 bacterial pathogens were isolated. The bacterial isolates comprised of *Campylobacter* species, 20 (12.7%), *Shigella* species, 11 (7.0%), and *Salmonella* species, 4 (2.5%) indicating that diarrheagenic pathogens other than *H. pullorum* are the main etiologic agents of diarrhea in children (Mulatu et al., 2014). Therefore, the evidence of the bacterium's association with diarrheal disease is weak; however it seems likely that Crohn's disease and cholelitiasis have more significant associations with *H. pullorum* infection. This association is not surprising since the bacterium along with *Helicobacter bilis* is able to tolerate high bile stress and is supported by several reports from Germany, Sweden, China, and Japan suggesting *H. pullorum* prevalence of 2–27% in gall bladder malignancies (Fukuda et al., 2002; Murata et al., 2004; Bohr et al., 2007; Chen et al., 2007; Karagin et al., 2010). Meanwhile a study in Chile and another from Ukraine report much higher prevalence rates (Fox et al., 1998; Apostolov et al., 2005). *H. pullorum* has also been found to be the predominant *Helicobacter* species in patients with Crohn's disease (Young et al., 2000; Bohr et al., 2004).

## Experimental infection model

EHS, including *H. pullorum* and *H. pullorum*-like living organisms, have been found to bring about bacteraemia and systemic ailment in both immunocompromised and immunocompetent patients. As described earlier, *H. pullorum* identified by PCR tests has been associated with enteric and hepatobiliary illnesses in humans. In comparison to different EHS, *H. pullorum* is viewed as an emerging, zoonotic human pathogen justifying the need to create animal models in order to understand the underlying pathogenic mechanisms. The bacterium possesses broad host specificity with the ability to infect birds, rodents, and humans.

Routine observation testing at a business rat creation office distinguished *Helicobacter* infected animal groups by PCR in BN/MolTac rats as well as C57BL/6NTac, C3H/HeNTac, and DBA/2NTac mice. Of the 10 C57BL/6NTac mice, 8 of 10 caecal and seven of 10 colon samples were PCR positive for *Helicobacter sp*. Only the caecum from one of three C3H/HeNTac mice was positive for *H. pullorum* (Turk et al., 2012). *H. pullorum* was also reported to be the causative agent of an outbreak in C57BL/6NTac and C3H/HeNTac mice housed within one isolated barrier unit. The isolates were phylogenetically similar to a human isolate, depicting a shift in host specificity (Boutin et al., 2010). The importance of *H. pullorum* in clinical ailment requires further studies to establish causative link. C57BL/6NTac mice can be persistently infected with *H. pullorum* in experimental settings providing the opportunity to utilize a mouse model to study *H. pullorum* pathogenesis (Turk et al., 2012).

## Pathogenesis

*H. pullorum* shows similarity with other *Helicobacter* species and *Campylobacter* species with regards to presence of several virulence factors. It has been isolated from patients suffering from cholecystitis, liver problems and cirrhosis (Ponzetto et al., 2000; Ananieva et al., 2002). Therefore, involvement of *Helicobacter* spp. including *H. pullorum* in pathogenesis and progression of cirrhosis, particularly in HCV-infected individuals seems plausible (Ponzetto et al., 2000). It has been also found that *H. pullorum* like organisms are present in individuals suffering from bacteremia, especially those who were immunocompetent. Later, association of *H. pullorum* infection with inflammatory bowel disease (IBD) has been speculated (Jamshidi et al., 2014).

Many virulence factors aid in the pathogenicity of the host cell by the bacterium including the bacterial flagellar apparatus, T3SS secreted toxin Cdt and the recently described T6SS. The cytopathogenic alterations induced by several human and avian *H. pullorum* strains were investigated on human intestinal epithelial cell lines. Human hepatocytes, gall bladder epithelial cells, and colon epithelial cells infected with *H. pullorum*, showed increased expression of MMP-2 and MMP-9 compared to uninfected controls in a bacterial dose dependent manner. These matrix metalloproteinases (MMPs) aid in degradation of extracellular matrix, allowing bacteria to interact with host cells (Yanagisawa et al., 2005).

*H. pullorum* is able to interact with host intestinal microvilli via its flagellum. This flagellum-microvilli interaction stimulates IL-8 production and intestinal cell colonization. This bacterial invasion process cause host damage via cellular edema and cell debris release (Sirianni et al., 2013). Recently it has been reported that the secretion of IL-8 leading to inflammatory reponses in gastric epithelial cells is dependent upon bacterial attachment to the epithelial lining (Sirianni et al., 2013). This inflammation is enhanced by cytolethal distending toxin (CDT) and lipopolysaccharide (LPS) via activation of the NF-kB pathway (Ceelen et al., 2005a).

More recently it has been observed that the bacterium is able to induce nitric oxide production in murine macrophages after internalization. The interaction of *H. pullorum* with host macrophages also stimulates secretion of pro-inflammatory cytokines TNF-α, IL-1β, IL-6, and MIP-2 (Parente et al., 2016).

## Virulence determinants

**Figure 1:**
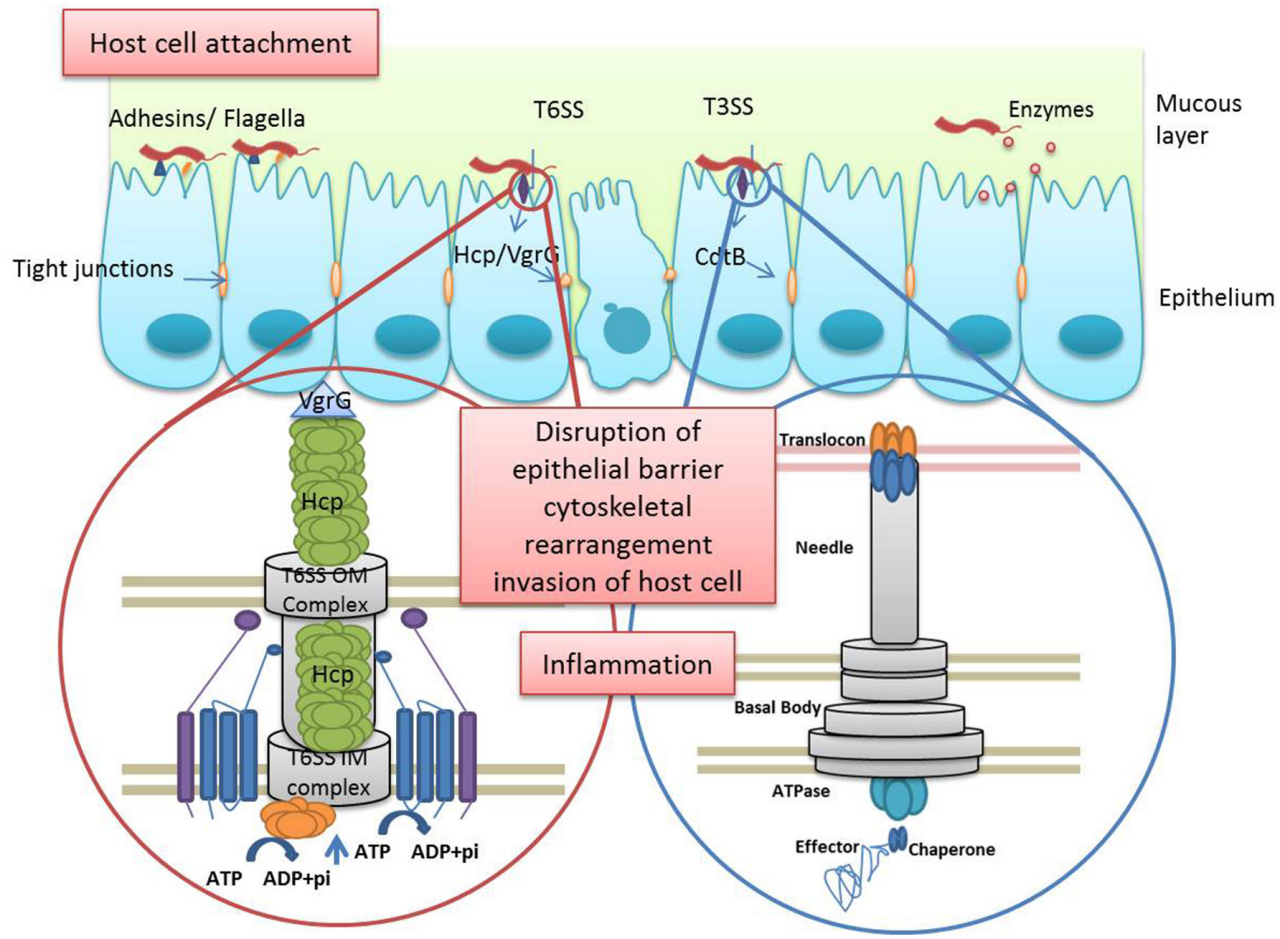
**Schematic representing major *H. pullorum* virulence factors**. *H. pullorum* virulence factors aid in the pathogenecity and colonization of the host cell by the bacterium including the cell-binding factor 2, flagellin, as well as type 6 secretion proteins Hcp and VgrG. The polar flagellum mediates initial contact with host cells via a flagellum–microvillus interaction. This contact leads to injection of CdtB toxin in the host cell which along with the newly described T6SS is involved in cell invasion and cytoskeletal rearrangement of host cell leading to epithelial barrier disruption. The inflammation elicited by the bacterium is enhanced by cdt and LPS.

*H. pullorum* causes gastroenteritis in poultry as well as humans; however few mechanisms of bacterial pathogenesis and its molecular determinants have so far been characterized. Following we list a few bacterial virulence factors described in *H. pullorum* (Summarized in Figure 1).

### Adhesins

Bacterial co-culture experiments with the mammalian intestinal epithelial cell line, Caco-2 showed that *H. pullorum* is capable of host cell adhesion, albeit at a much lesser extent in comparison to *Salmonella typhimurium* and comparable to the adhesion rates observed for *C. jejuni* (Varon et al., 2009; Sirianni et al., 2013). Factors responsible for cytopathogenic effects of *H. pullorum* on epithelial cells have not been formally identified. However, cell-binding factor 2, flagellin, secreted protein Hcp, valine-glycine repeat protein G (VgrG), a type VI secretion protein and a protease were identified as important virulence and colonization factors in *H. pullorum* (Sirianni et al., 2013). Furthermore, scanning electron microscopy suggests that the polar flagellum of *H. pullorum* mediates initial contact with host cells via a flagellum–microvillus interaction and that host cell contact is important for inflammation elicited by the bacterium (Sirianni et al., 2013; Varon et al., 2009).

### Cdt (cytolethal distending toxin)

Cytolethal distending toxin (Cdt) was first reported by Jhonson and Lior in *E. coli* in the year 1987 and was described to cause cellular anomalies and cell death in Chinese Hamster ovary (Ceelen et al., 2006b). Cdt causes edema, cytoskeleton anomalies and G2/M cycle arrest in host cell. Cdt has been identified in *Campylobacter* species as well as in different *Helicobacter* species like *H. bilis, H. canis, H. hepaticus*, and *H. pullorum* (Mohamed et al., 2010). *Two* soluble factors involved in cytotoxic activity were reported in *H. pullorum*: the Cdt toxin and a soluble toxic factor, still unidentified, causing a mitotic catastrophe resulting in primary necrosis of hepatic cells. CdtB induced a cellular and nuclear enlargement, accompanied by profound remodeling of the actin cytoskeleton with the formation of cortical actin-rich large lamellipodia and membrane ruffle structures. In addition, disturbance of focal adhesion and the microtubule network were also observed. These effects may have profound consequences on bacterial adherence and intestinal barrier integrity (Varon et al., 2014). Therefore, *H. pullorum* Cdt is responsible for major cytopathogenic effects *in vitro*, confirming its role as an important virulence factor of this emerging human pathogen (Young et al., 2000).

### Type 6 secretion system (T6SS)

T6SS is a newly identified secretion system in gram negative bacteria encoded in pathogenicity islands (Bingle et al., 2008). The T6SS is composed of 13 core components and displays structural similarities with the tail-tube of bacteriophages. The phage uses a tube and a puncturing device to penetrate the cell envelope of target bacteria and inject DNA. It is proposed that the T6SS creates a specific path in the bacterial cell envelope to drive effectors and toxins to the surface. T6SS device can also perforate other cells with which the bacterium is in contact, thus injecting the effectors into these targets. The tail tube and puncturing device of the T6SS are composed of *Hcp* and *VgrG* proteins, respectively (Hachani et al., 2013). *Hcp* and *VgrG* are T6SS effector proteins, the presence of which is considered a prerequisite for T6SS function. Both *Hcp* and *VgrG* are extracellular components forming a needle like projection that makes contact with the host cell. Hcp forms a hexametric ring that is believed to stack into a tubular structure. On the other hand, VgrG proteins can in fact have an effector function. So called evolved *VgrG* has a sizeable C-terminal containing effector domain (Pukatzki et al., 2007). The existence of a putative functional T6SS in *H. pullorum* was proposed by Sirianni in 2013 (Sirianni et al., 2013). Genomic analysis of three out of four chicken isolates depicted the presence of T6SS genes (Borges et al., 2015). Certain structural similarities between Hcp and endocytic vesicle coat proteins suggest its role in cellular invasion via interaction with endocytic vesicles of host, although *in vitro* validation of this *in silico* study is lacking. In addition, the T6SS is associated with a more severe form of diarrhea and bacteremia during *C. jejuni* infection supporting the contribution of the system to the virulence of this pathogen (Bleumink-Pluym et al., 2013).

It has been shown that the T6SS effector proteins, vgrG and Hcp also play a major role in host cell pathogenesis, although their role in *H. pullorum* infection has not been completely understood. VgrG proteins form a trimeric, needle-like structure and puncture host cell membrane. Hcp is involved in induction of actin cytoskeleton rearrangement and production of IL-6 and IL-8. Furthermore, two proteins suggested to be 1-phosphatidylinositol-4- phosphate 5-kinases were found to be involved in the regulation of the actin cytoskeleton and were also identified in proximity to the T6SS proteins (Sirianni et al., 2013).

## Immune response and immunogenic proteins

Immunogenic cell surface proteins of *H. pullorum*, along with other EHS compared to *H. pylori* were characterized via two dimensional electrophoresis and immunoblotting using immunized rabbit antisera. Twenty-One specific immunogenic proteins were identified, with proteins of *H. pylori* and *H. pullorum* showing similarities in their protein profiles (Kornilovs'ka et al., 2002).

## Antibiotic resistance and resistance mechanisms

Although *H. pullorum* infection has been associated with gastroenteritis and vibrionic hepatitis, there is no antibiotic recommendation for this organism. Isolates of poultry origin show resistance to ciprofloxacin, gentamycin, erythromycin and tetracyclin and is susceptible to colistin sulfate and ampicillin (Hassan et al., 2014a). Draft genome sequence of *H. pullorum* human isolate, MIT 98-5489 reveal that the bacteria are clarithromycin resistant. This resistance may be mediated by a mutation in the 23S rRNA gene. On the other hand, Rifampin resistance is conferred by four missense mutations in *RpoB*. *H. pullorum* (MIT 98-5489) is also resistant to ciprofloxacin. This is consistent with the finding that individual missense mutations were detected in *gyrA* which is responsible for conferring ciprofloxacin resistance (Shen et al., 2014). Furthermore, a triple-base-pair mutation in 16S rRNA is reported to confer tetracyclin resistance to the bacterium as well (Borges et al., 2015).

On the other hand *H. pullorum* human isolate (16S rRNA sequence accession no. AF334681) shows susceptibility to aminoglycosides and third-generation cephalosporins, β-lactams, and doxycycline (Tee et al., 2001). Keeping this in mind treatment strategies for patients may be recommended. The antibiotic susceptibility may vary according to strain, especially in geographically distinct regions. Intensive farming practices, where antibiotics are routinely fed to livestock as growth promoters and to prevent potential bacterial infections have contributed to increase in drug resistance worldwide, enabling re-emergence of zoonotic infections (Andersson, 2003). Most of these antibiotics have been banned in the European Union which may predict a very different antibiotic susceptibility pattern in isolates from other regions (Roe and Pillai, 2003; Cantas et al., 2013).

## Conclusion

Zoonotic pathogens are twice as likely to be associated with emerging zoonosis including approximately 12% of human pathogens (Taylor et al., 2001). According to WHO, emerging or reemerging zoonosis are diseases caused by novel or partially new etiological agents or by a microorganism previously known but now occurring in species or places where the disease was unknown (Meslin, 1992). Engering and colleagues describe a comprehensive framework of disease emergence depicting drivers of pathogen emergence including (i) its description in a novel host; (ii) occurrence of a mutant pathogen with novel traits with ability to cause more severe form of the disease; or (iii) presence in a novel geographic region. These factors change the overall pattern of the pathogen–host–environment interactions leading to disease emergence (Engering et al., 2013). Presence of *H. pullorum* in various geographical zones as well as its wide range of hosts may pose a potential health risk. This is further confounded by the *H. pullorum* outbreak reported in mice raised in a barrier facility (Boutin et al., 2010).

Recent epidemiological data shows Salmonellosis and Campylobacteriosis to be the most frequent food-borne bacterial zoonoses in Europe (team Ee, 2013). It is unknown whether *H. pullorum* in humans is acquired by eating uncooked poultry, as is the case with *C. jejuni* acquired zoonotic infection. However, human transmission from poultry seems likely, considering the high prevalence rates reported from various regions. Routine surveillance of the pathogen in poultry as well as clinical samples is necessary. Future studies determining common sequence types in isolates of human and poultry origin as well as description of specific source markers will enable source tracking and infection risk in the population.

## Author contributions

FG, KJ, and SJ contributed to the literature review and writing of the manuscript. SJ edited the manuscript and approved final draft. HB contributed to the final edit and critical review of the manuscript.

### Conflict of interest statement

The authors declare that the research was conducted in the absence of any commercial or financial relationships that could be construed as a potential conflict of interest.
